# A Case of Sinonasal Actinomycosis Associated With an Odontogenic Cyst of Ectopic Dental Origin

**DOI:** 10.7759/cureus.88209

**Published:** 2025-07-17

**Authors:** Kana Nanjo, Ryoji Kagoya, Munetoshi Hinata, Kento Koda, Kyohei Horikiri, Hironobu Nishijima, Kenji Kondo

**Affiliations:** 1 Department of Otorhinolaryngology and Head and Neck Surgery, Graduate School of Medicine, The University of Tokyo, Tokyo, JPN; 2 Department of Pathology, Graduate School of Medicine, The University of Tokyo, Tokyo, JPN

**Keywords:** actinomycosis, antibiotics, ectopic tooth, endoscopic sinus surgery, odontogenic cyst

## Abstract

Actinomycosis rarely occurs in the sinonasal region. Here, we report a case of sinonasal actinomycosis associated with an odontogenic cyst of ectopic dental origin. A 28-year-old female patient was referred to our department with a complaint of left buccal pain. Nasal endoscopy revealed a polypoid mass with purulent discharge in the left nasal cavity. Contrast-enhanced computed tomography revealed a mass lesion occupying the left maxillary sinus and nasal cavity, along with an ectopic tooth adjacent to the lesion. T2-weighted magnetic resonance imaging showed a mass lesion with a low-intensity signal. We performed endoscopic sinus surgery and resected the mass lesion and the ectopic tooth. Histopathological examination revealed the presence of *Actinomyces* species. No tissue-invasive *Actinomyces* were detected in the resected tissues. At the two-year follow-up, no recurrence of actinomycosis was observed despite the absence of antibiotic therapy. To our knowledge, there have been no reports of actinomycosis associated with odontogenic cysts of ectopic dental origin. Although surgery followed by antimicrobial therapy is generally recommended for actinomycosis, sinus surgery alone may have a sufficient bactericidal effect against *Actinomyces* by exposing the lesion to the open air. The clinical course of this case supports the notion that postoperative antibiotic therapy is not mandatory for patients with sinonasal actinomycosis.

## Introduction

*Actinomyces* are Gram-positive, filamentous, anaerobic bacteria that colonize the oral cavity, gastrointestinal tract, and urogenital tract [1]. Actinomycetes sometimes invade the submucosal tissue following mucosal injury and cause chronic granulomatous infections, which are known as actinomycosis [2]. *Actinomyces israelii* is the species that most frequently causes infections [3]. Although 50%-60% of actinomycosis cases occur in the head and neck region, involvement of the sinonasal region is relatively rare [4,5]. Approximately half of actinomycosis cases in the head and neck region occur in the mandible [4]. Therefore, information on the clinical features and management strategies of sinonasal actinomycosis remains limited, even though surgery followed by antibiotic treatment is recommended [4]. Here, we report a case of sinonasal actinomycosis associated with an odontogenic cyst of ectopic dental origin.

## Case presentation

**Figure 1:**
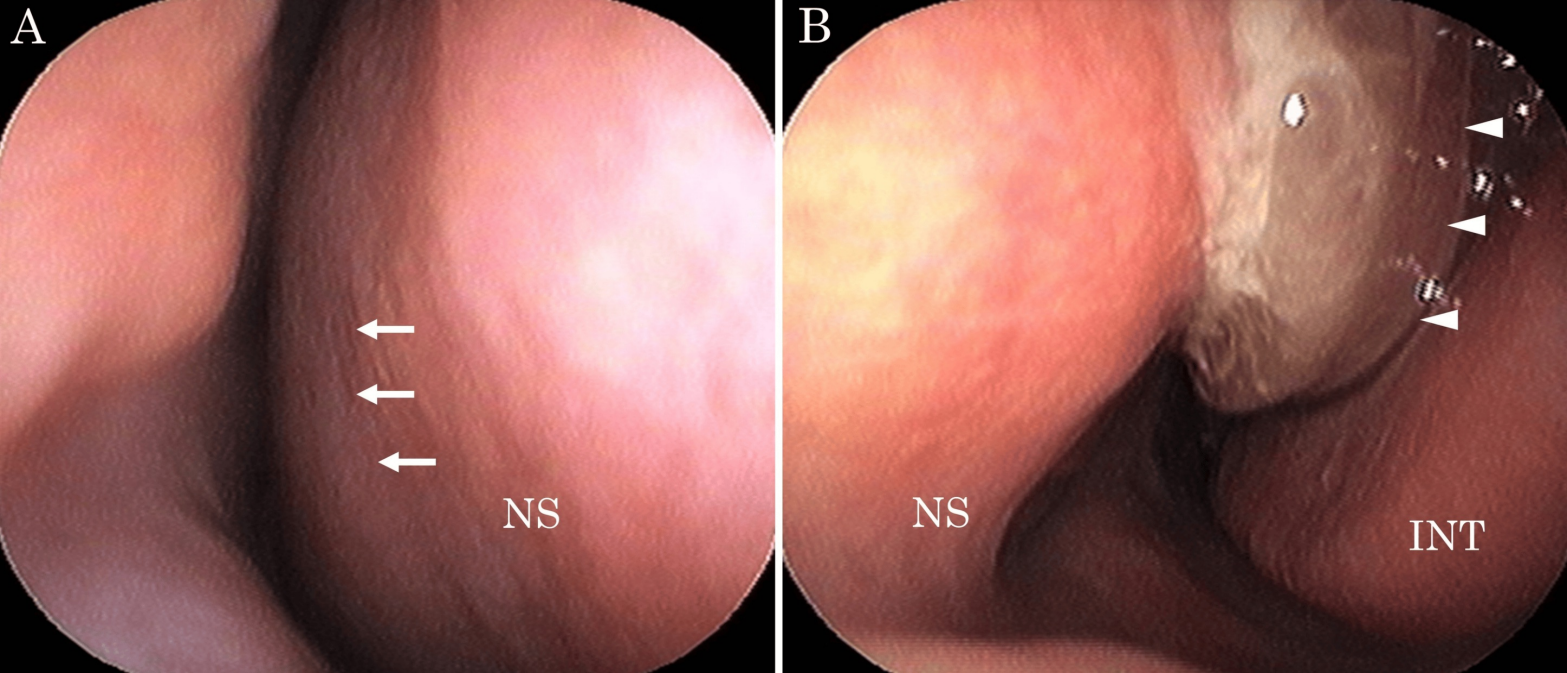
Image of nasal endoscopy at the first visit (A) The right nasal cavity is narrowed by a deviated nasal septum (arrow) caused by a mass lesion in the left nasal cavity. (B) A polypoid mass with purulent discharge occupying the left common nasal meatus is observed (arrowhead). INT: inferior nasal turbinate; NS: nasal septum

**Figure 2:**
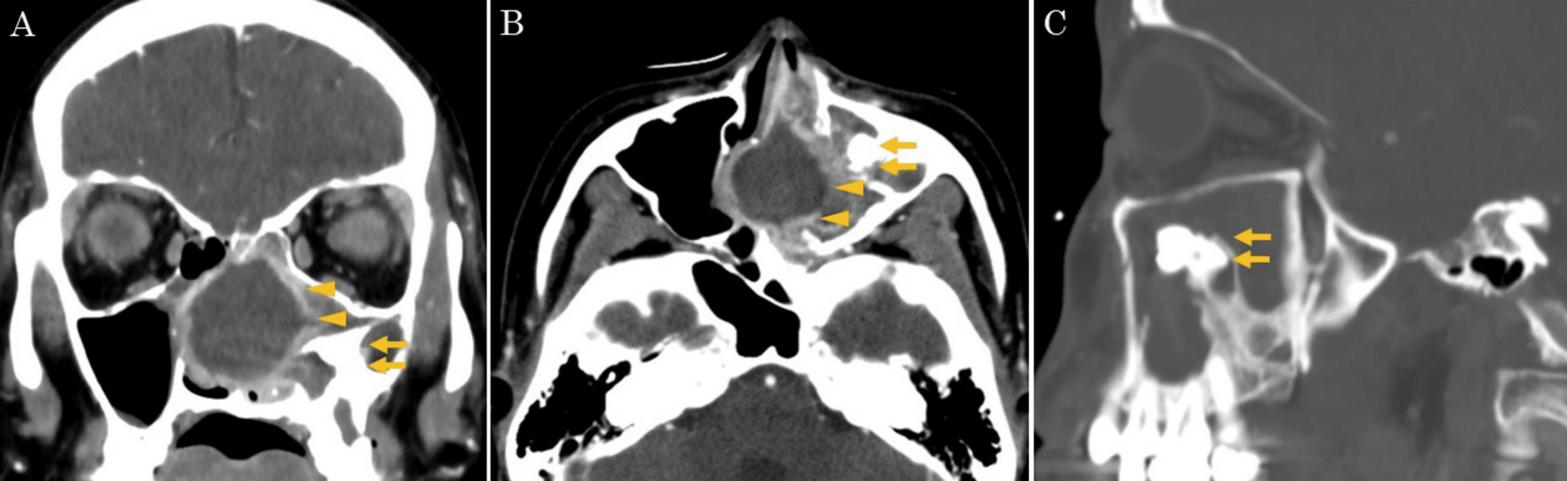
Contrast-enhanced sinus CT images at the first visit Coronal (A) and axial (B) images show a mass lesion with enhanced margins in the left nasal cavity and maxillary sinus (arrowhead) and an ectopic tooth adjacent to the lesion (arrow). The perpendicular plate of the ethmoid bone is destroyed. Sagittal CT in the bone window image (C) show the ectopic tooth eruption in the floor of the maxillary sinus. CT: computed tomography

**Figure 3:**
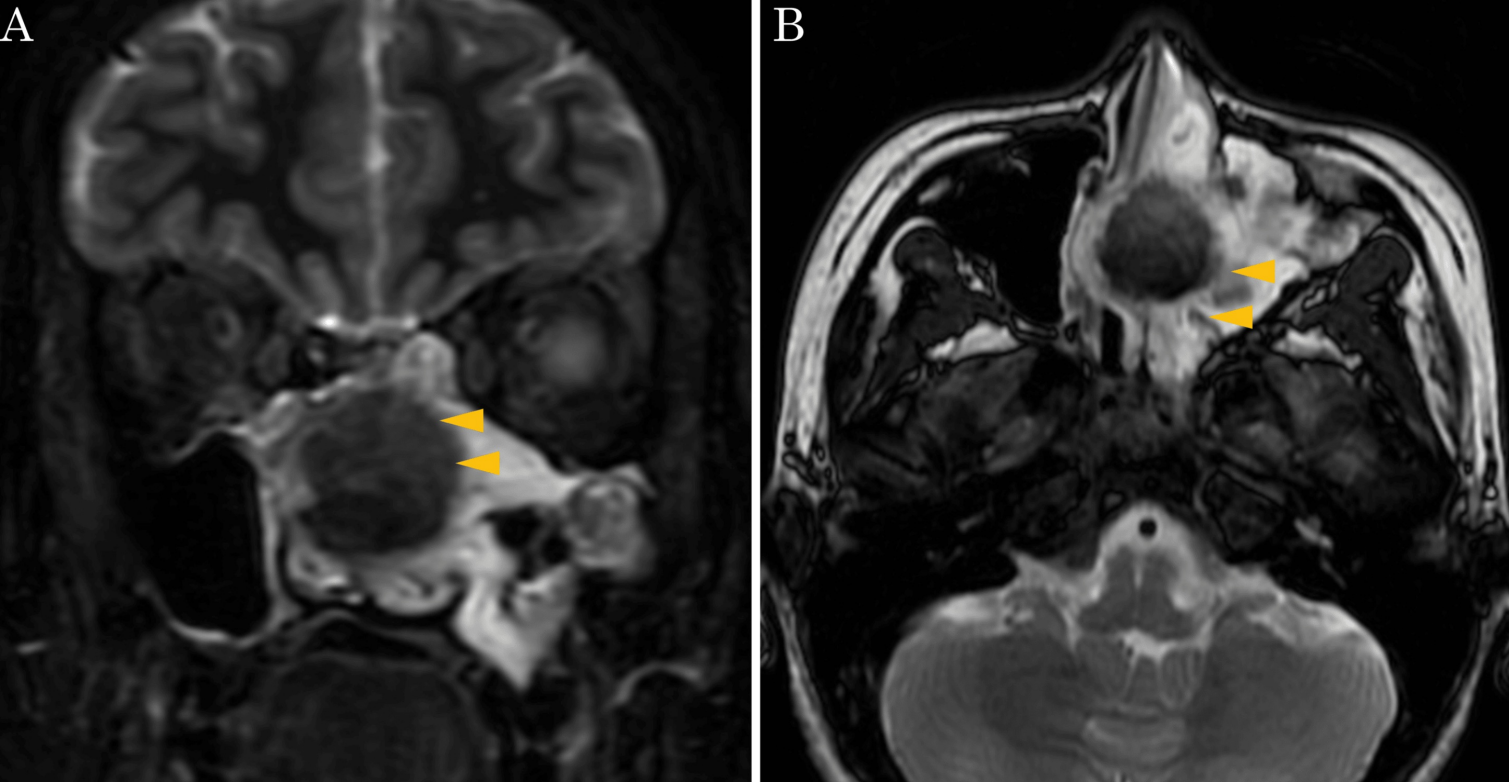
Sinus MRI Coronal (A) and axial (B) T2-weighted images show a mass lesion with low-intensity signals (arrowhead) in the left nasal cavity and maxillary sinus. MRI: magnetic resonance imaging

A 28-year-old woman with a history of Kawasaki disease presented to a local dental and otolaryngology clinic with the chief complaint of persistent left buccal pain for several years. She had no coronary artery aneurysm caused by Kawasaki disease and was not taking anticoagulant medication. Left maxillary sinusitis was suspected, and the patient was referred to our hospital for further evaluation. Nasal endoscopy revealed a deviated nasal septum and a polypoid mass with purulent discharge occupying the left common nasal meatus (Figure 1). Contrast-enhanced computed tomography (CT) of the sinus revealed a mass lesion with an enhanced margin in the left maxillary sinus extending to the nasal cavity, along with an ectopic tooth adjacent to the lesion (Figure 2). The perpendicular plate of the ethmoid bone was destroyed. T2-weighted magnetic resonance imaging (MRI) showed a mass lesion with a low-intensity signal (Figure 3).

**Figure 4:**
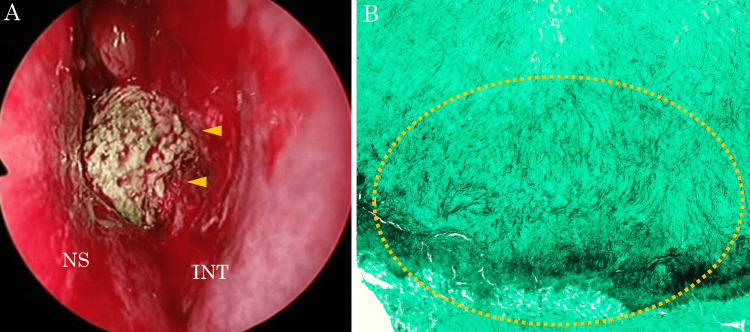
Intraoperative and histopathological images (A) Intraoperative endoscopic image of the left nasal cavity. Caseous material within the mass lesion is visible (arrowhead). (B) The Grocott-stained section of the material reveals *Actinomyces* (within dotted circle). INT: inferior nasal turbinate; NS: nasal septum

**Figure 5:**
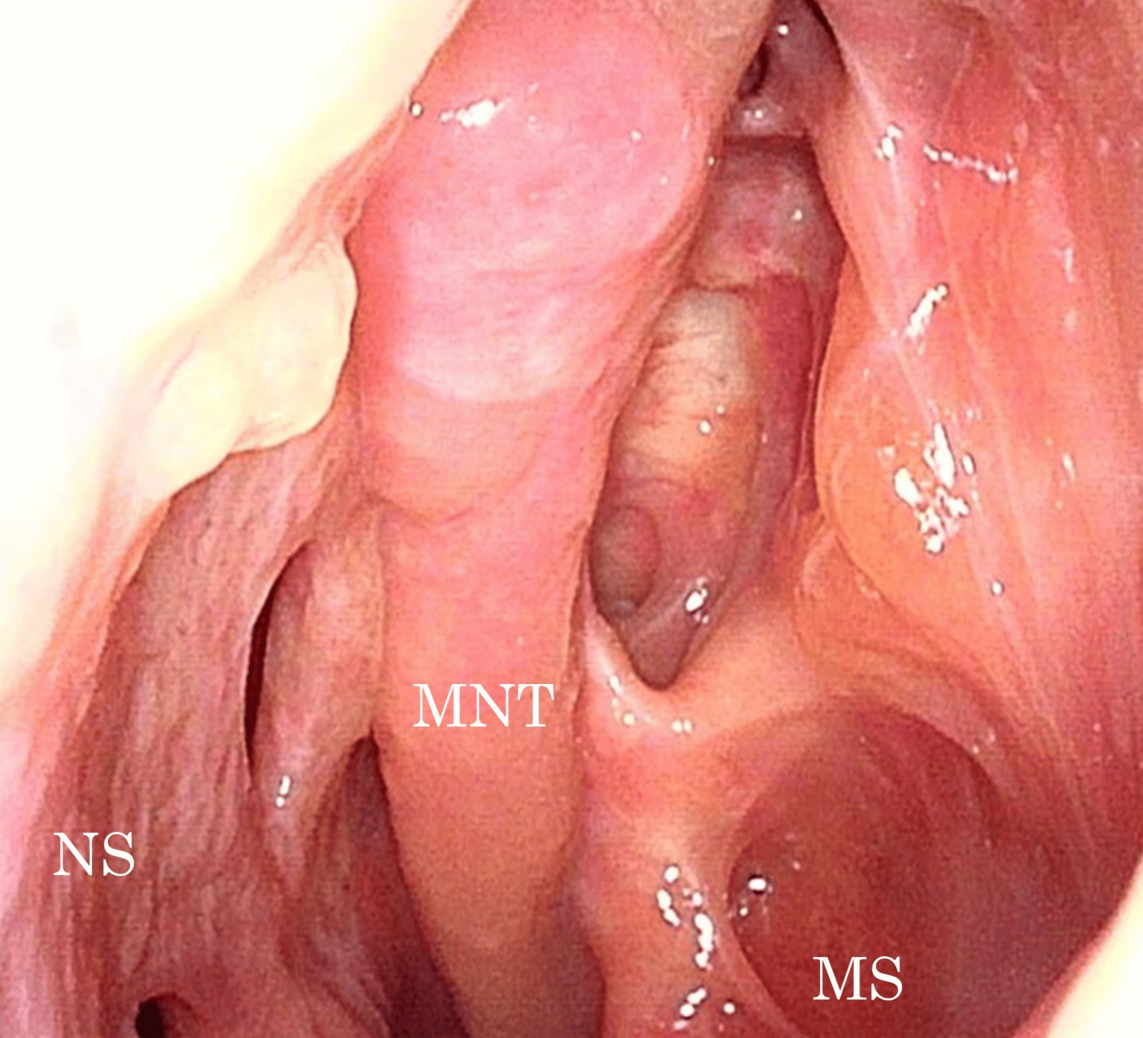
Nasal endoscopy image two years after surgery Nasal endoscopy reveals no recurrence of actinomycosis. MNT: middle nasal meatus; MS: maxillary sinus; NS: nasal septum

Based on these findings, an odontogenic cyst associated with an ectopic tooth was suspected. Endoscopic sinus surgery (ESS) was performed based on diagnosis and as treatment. Intraoperatively, caseous material filling the mass lesion occupying the left nasal cavity was observed (Figure 4A). The mass lesion had destroyed a significant portion of the nasal septal cartilage and the perpendicular plate of the ethmoid bone. However, most of the nasal septal mucosa remained, with only two small perforations in the posterior nasal septum. The ectopic tooth erupted from the floor of the maxillary sinus toward the anterior direction. We resected the mass lesion and ectopic tooth using a combination of approaches via the natural ostium of the maxillary sinus and endoscopic modified medial maxillectomy. Highly thickened mucosa in the maxillary sinus was removed with a shaver. The mass lesion was considered to be an odontogenic cyst because it was adjacent to the ectopic tooth. Histopathological examination of the caseous material revealed the presence of actinomycetes (Figure 4B). No tissue-invasive actinomycetes were observed in resected tissues. At the two-year follow-up, no recurrence of actinomycosis was observed despite antibiotic treatment (Figure 5).

## Discussion

Actinomycosis is a pyogenic and granulomatous infection. It is most commonly found in the head and neck (40%-55%), chest (15%-35%) and abdomen (10%-20%) [6]. In the head and neck region, actinomycosis most frequently occurs in the mandible [4]. Damage to oral mucosal epithelial cells resulting from poor oral hygiene, dental disease or trauma may contribute to the development of actinomycosis in the mandibular region [7]. The sinonasal region is slightly farther away from the oral cavity than the mandible and is separated by multiple layers of bony partitions, which may make it less susceptible to the spread of actinomycosis. Pathologically, actinomycetes are branching filamentous organisms stained black with Grocott methenamine silver and often accompanied by sulfur granules [4]. Actinomycosis of the paranasal sinuses is relatively rare, although approximately 20 case reports have been reported [4-15]. Sinus actinomycosis is presumed to be caused by the migration of oral pathogens into the sinuses due to dental fistulae or maxillofacial trauma [10].

There has been only one report each of sinus actinomycosis associated with an ectopic tooth [16] and odontogenic cyst-derived actinomycosis [17] to date. Zalagh et al. reported a case of actinomycosis with an ectopic tooth in the common nasal meatus successfully removed under local anesthesia. There was no destruction of the sinonasal tissues [16]. Hwang et al. reported six cases of odontogenic cyst-derived actinomycosis treated by enucleation or curettage. Although no information on CT or MRI findings was available, the average diameter of the cysts was 11.7 mm and none of the cases required treatment of the paranasal sinuses [17]. Compared with these previous reports, the present case is considered to be more severe because the sinonasal structures were destroyed. To our knowledge, there have been no previous reports of actinomycosis associated with odontogenic cysts of ectopic dental origin. Surgical excision of lesions and postoperative antimicrobial treatment are generally recommended for patients with actinomycosis [5,11]. Therefore, surgeons should consider the potential presence of actinomycosis when removing ectopic teeth or odontogenic cysts from the sinuses to ensure accurate histopathological diagnosis and appropriate treatment.

Although ESS followed by antimicrobial therapy for several months to a year is recommended for the treatment of sinonasal actinomycosis [5,11], some reports suggest that the postoperative long-term antimicrobial therapy may not be essential in cases of sinonasal actinomycosis. Numano et al. [4] and Woo et al. [9] suggested that low-dose macrolides administered for two or three months might be adequate for the postoperative treatment of sinonasal actinomycosis without tissue invasion. Unlike the subcutaneous tissues of the head and neck, the paranasal sinuses can be adequately exposed to the open air by ESS; therefore, the increased oxygen concentration at the infected site has a bactericidal effect on anaerobic bacteria, including actinomycetes [18]. In the present case, tissue-invasive actinomycetes were not observed in the resected mucosal tissues. Therefore, we did not perform postoperative antimicrobial treatment after consultation with the patient. The patient has remained recurrence-free for two years postoperatively. The clinical course of the present case supports the hypothesis that postoperative antimicrobial treatment for sinonasal actinomycosis is not mandatory.

The typical CT findings of sinonasal actinomycosis have been reported to include calcification and bone destruction [9], while little is known about the MRI findings. In this case, T2-weighted MRI revealed a lesion with a low-intensity signal. Only two case reports of sinonasal actinomycosis with preoperative MRI evaluation have been reported [8,13]. In the report, as in our case, the actinomycete mass showed a low-intensity signal on T2-weighted MRI [8]. The low-intensity signal of fungal balls on T2-weighted MRI has been reported to be caused by metals, such as iron, magnesium, and manganese [19]. The metabolites of actinomycetes may also contain large amounts of these metals. To improve the accuracy of preoperative diagnosis, it is important to accumulate knowledge of MRI findings in sinonasal actinomycosis.

## Conclusions

We reported a rare case of sinonasal actinomycosis associated with an odontogenic cyst of ectopic dental origin. This case report adds to the current knowledge by suggesting that ectopic odontogenic cysts can be associated with actinomycosis and that postoperative antimicrobial treatment for sinonasal actinomycosis may not be mandatory.
